# Supplementary material for: Brassinosteroids Induce Strong, Dose-Dependent Inhibition of Etiolated Pea Seedling Growth Correlated with Ethylene Production
Source: Biomolecules. 2019 Dec 9;9(12):849. doi: 10.3390/biom9120849 (PMC7017382; doi:10.3390/biom9120849)
Supplement: Supplementary file 1 [file biomolecules-09-00849-s001.pdf]

Supplementary data

BRASSINOSTEROIDS INDUCE STRONG, DOSE-DEPENDENT INHIBITION OF ETIOLATED PEA SEEDLING GROWTH CORRELATED WITH ETHYLENE PRODUCTION

Petra Jiroutová, Jaromír Mikulík, Ondřej Novák, Miroslav Strnad and Jana Oklestkova*

Laboratory of Growth Regulators, The Czech Academy of Sciences, Institute of Experimental Botany & Palacký University, Faculty of Science, Šlechtitelů 27, 78371 Olomouc, Czech Republic.

**Corresponding author:* E-mail address: jana.oklestkova@upol.cz, Tel. +420585634853 (J. Oklestkova)

SuppTable1 statistical data for Fig. 4 – length of epicotyls after treatment, *represents p value <0.05, **represent p value <0.01, ***represent p value <0.001.

ttest	compound	c [mol/L]	p value		ttest	compound	c [mol/L]	p value	
	BL	2,00E-09	0,51911	-		IAA	2,00E-09	0,360875	-
		2,00E-08	0,522542	-			2,00E-08	0,747311	-
		2,00E-07	0,325809	-			2,00E-07	0,161763	-
		2,00E-06	0,002028	**			2,00E-06	0,414765	-
		2,00E-05	2,54E-05	***			2,00E-05	0,918297	-
		2,00E-04	1,64E-09	***			2,00E-04	0,001709	**
		2,00E-03	4,96E-10	***			2,00E-03	2,25E-06	***
		2,00E-02	3,35E-10	***			2,00E-02	3,82E-10	***
	24epiBL	2,00E-09	0,087668	-		GA3	2,00E-09	0,695175	-
		2,00E-08	0,084818	-			2,00E-08	0,017927	*
		2,00E-07	0,829234	-			2,00E-07	0,009965	**
		2,00E-06	0,526198	-			2,00E-06	0,015807	*
		2,00E-05	6,07E-09	***			2,00E-05	0,00052	***
		2,00E-04	3,46E-09	***			2,00E-04	0,000427	***
		2,00E-03	3,88E-11	***			2,00E-03	0,000324	***
		2,00E-02	5,02E-12	***			2,00E-02	0,001915	**
	tZR	2,00E-09	0,318369	-		TDZ	2,00E-09	0,571013	-
		2,00E-08	0,449671	-			2,00E-08	0,009998	**
		2,00E-07	0,847271	-			2,00E-07	0,856863	-
		2,00E-06	0,6008	-			2,00E-06	0,000577	***
		2,00E-05	0,229864	-			2,00E-05	0,069406	-
		2,00E-04	0,929079	-			2,00E-04	0,07741	-
		2,00E-03	0,365523	-			2,00E-03	3,88E-06	***
		2,00E-02	0,04463	*			2,00E-02	8,11E-07	***
